# Regulation of Ferroptosis in Human Macrophage by Nitric Oxide Donors

**DOI:** 10.17691/stm2025.17.3.04

**Published:** 2025-06-30

**Authors:** I.I. Vlasova, M.D. Yurkanova, A.A. Zolotopup, T.O. Klyucherev, P.S. Timashev

**Affiliations:** Leading Researcher, Department of Modern Biomaterials; I.M. Sechenov First Moscow State Medical University (Sechenov University), 8/2 Trubetskaya St., Moscow, 119991, Russia; Laboratory Assistant, Laboratory of Clinical Smart Nanotechnologies; I.M. Sechenov First Moscow State Medical University (Sechenov University), 8/2 Trubetskaya St., Moscow, 119991, Russia; Laboratory Assistant, Laboratory of Clinical Smart Nanotechnologies; I.M. Sechenov First Moscow State Medical University (Sechenov University), 8/2 Trubetskaya St., Moscow, 119991, Russia; Junior Researcher, Laboratory of Clinical Smart Nanotechnologies; I.M. Sechenov First Moscow State Medical University (Sechenov University), 8/2 Trubetskaya St., Moscow, 119991, Russia; Professor, Institute of Regenerative Medicine; Scientific Director, Scientific and Technological Park of Biomedicine; I.M. Sechenov First Moscow State Medical University (Sechenov University), 8/2 Trubetskaya St., Moscow, 119991, Russia

**Keywords:** ferroptosis, THP-1 macrophages, nitric oxide donors

## Abstract

Ferroptosis is a programmed form of cell death in which iron-dependent lipid peroxidation is the main feature. Macrophages are the major cells of the immune system, they function in a pro-oxidative environment, so the study of their susceptibility to ferroptosis and the search for approaches to its regulation are important.

**The aim of the study** was to investigate ferroptosis in macrophages differentiated from THP-1 myeloid leukemia cells and to compare the effect of NO donors with different half-lives on the degree of ferroptosis development.

**Materials and Methods:**

RSL3 and ML-162, inhibitors of glutathione peroxidase 4 (GPX4), and erastin, an inhibitor of cystine/ glutamate transport, were used to induce ferroptosis in THP-1 macrophages. The progression of ferroptosis was monitored using three independent methods: reduction of Alamar blue by live cells, measurement of lactate dehydrogenase in the medium, and the LIVE/DEAD assay. Ferroptotic cell death was proven by using the specific inhibitor ferrostatin-1 and by detecting lipid oxidation in cells using the BODIPY 581/591 C11 fluorescent probe.

**Results:**

RSL3 and ML-162 dose-dependently induced ferroptosis in cells. THP-1 macrophage ferroptosis is a slow process and begins ~5 h after inducer addition. Erastin was a weak ferroptosis inducer; however, it enhanced ferroptosis induced by GPX4 inhibitors. We compared the ability of two NO donors with different half-lives to affect THP-1 macrophage ferroptosis: DEA NONOate (2 min) and DTPA NONOate (3 h). Donors were added either once after the inducer at a concentration of 100–120 μM or repeatedly until reaching the final concentration. DEA had no effect on THP-1 macrophage ferroptosis, whereas DPTA completely inhibited ferroptosis.

**Conclusion:**

DTPA, being an NO donor with a half-life of 3 h at 37°С, can be used to inhibit ferroptosis in THP-1 macrophages, which develops within 17–19 h. Therefore, there are mechanisms of prolongation of NO action in cells that should be studied to use NO donors for regulation of cellular ferroptosis.

## Introduction

Ferroptosis is a distinct type of regulated programmed cell death resulting from the disruption of three metabolic processes: intracellular glutathione concentration, iron metabolism, and the regulation of lipid peroxidation [1, 2]. Lipid peroxidation has been recognized as a fundamental component of ferroptosis [3, 4]. Currently, the association of ferroptosis with various pathological conditions is well established, ranging from neurodegenerative diseases and ischemia/reperfusion injuries to kidney disorders and therapy-resistant cancers [5–7]. This has stimulated the search for new mechanisms to modify the ferroptosis resistance in cells.

Glutathione peroxidase 4 (GPX4) catalyzes the reduction of phospholipid hydroperoxides (PLOOH) into their respective lipid alcohols (PLOH) via oxidation of thiol groups of glutathione (GSH) [8]. The GPX4 inhibition by specific RSL3 or ML-162 inhibitors serves as a standard experimental model for studying ferroptosis. As an alternative ferroptosis inducer, erastin, an inhibitor of a cystine/glutamate antiporter system Хc^–^, is widely used in laboratory research.

Macrophages are the major cells of the innate immune system participating in all stages of the immune response to tissue injury or pathogen exposure, as well as coordinating the actions of other immune cells. They produce pro-inflammatory cytokines and reactive oxygen species, creating a pro-oxidative environment that may contribute to the development of ferroptosis in cells [9–11]. THP-1 is a human monocytic leukemia cell line; activation of these cells by phorbol-12- myristate-13-acetate induces their differentiation into macrophage-like cells [12]. This cell line is a widely used model for studying the immunomodulatory properties of monocytes/macrophages both in fundamental immunology and as an *in vitro* model of various diseases involving macrophage-mediated pathogenesis (e.g., cardiovascular diseases, neuroinflammation, cancer, and infectious diseases) [13–15]. Although most studies on macrophage ferroptosis mechanisms have been conducted using murine macrophages — namely the RAW 264.7 cell line and bone marrow-derived macrophages — THP-1 cells have recently been actively studied in the context of diseases where cell death occurs via ferroptosis-dependent mechanisms. Elevated iron concentrations in endometriosis induce ferroptosis in THP-1 macrophages, resulting in reduced phagocytic capacity of these cells and increased secretion of angiogenic cytokines such as vascular endothelial growth factor A and interleukin 8. These factors contribute to the endometriosis progression [16]. Inhibition of ferroptosis in THP-1 macrophages by baicalein — a potential anti-ferroptosis agent due to its ability to upregulate GPX4 expression — restored phagocytic activity. This compound is considered a promising therapeutic agent for endometriosis treatment through attenuation of macrophage ferroptosis [16]. Therefore, THP-1 macrophages represent an important model for studying ferroptosis-dependent cell death, as well as for investigating pharmacological agents targeting the modulation of ferroptosis in macrophages.

GSH/GPX4 is the predominant system for phospholipid hydroperoxide detoxification in mammalian cells [8]. However, GPX4-independent ferroptosis regulators have recently been identified, such as ferroptosis suppressor protein 1 [17] and Ca^2+^- independent phospholipase A2β [18]. Several natural and synthetic free radical scavengers — ranging from vitamin E family compounds to various aromatic amines and phenolic compounds — act as inhibitors of ferroptosis [19]. It has been demonstrated that mouse RAW 264.7 macrophages can activate an anti-ferroptosis mechanism regulated by inducible NO synthase (iNOS) to block lipid peroxidation and protect cells from death [20]. Moreover, macrophages are capable of protecting not only themselves but also pulmonary epithelial cells in co-culture experiments [21].

Nitric oxide (NO^•^) is known to be a reactive molecule produced by the family of iNOS enzymes [22]. First, NO^•^ is a potent signaling molecule capable of paracrine action during smooth muscle relaxation or vasodilation, as well as functioning as an intracellular secondary messenger regulating cellular antioxidant responses [23]. NO^•^ directly binds and inactivates iron-containing enzymes and can react with superoxide radical anion to form highly reactive peroxynitrite, which participates in defense against pathogens [24]. Furthermore, NO^•^ ability to interact with various radicals, including intermediate lipid radicals generated during ferroptosis, may facilitate post-translational nitrosylation of proteins and lipids, thereby modulating their activity, stability, or localization. NO donors are compounds stable in organic solvents or in strongly alkaline pH conditions, where their stock solutions are prepared; however, when exposed to pH 7.4, they undergo disproportionation, releasing gaseous NO.

In the present study, we investigated ferroptosis in THP-1 macrophages and demonstrated a possibility of its regulation: enhancement by simultaneous action of inducers of different nature and inhibition by a nitric oxide donor.

## Materials and Methods

### Cell cultivation

Human monocytic THP-1 cells were cultured in RPMI 1640 medium without L-glutamine, with 2 mM GlutaMAX, 10% heat-inactivated fetal bovine serum (FBS), penicillin (100 U/ml), and streptomycin (100 μg/ml) at 37°C and 5% CO_2_. Differentiation into macrophages was induced by incubation with phorbol 12-myristate 13-acetate at 75 ng/ml for 48 h. After 2 days, the medium was replaced, and cells were cultured for an additional 24 h without phorbol 12-myristate 13-acetate followed by the addition of experimental compounds. For cell viability assays, cells were seeded in 48-well plates at a density of 150,000 cells per well in 350 μl of culture medium. Ferroptosis was induced using GPX4 inhibitors RSL3 and ML-162, which were added to the culture medium at concentrations of 0.5–2.5 μM, followed by incubation for 5–22 h and subsequent assessment of cell death. Additionally, the cystine/glutamate antiporter system inhibitor erastin was used to induce cell death. To confirm ferroptotic cell death, experiments were conducted using the ferroptosis inhibitor ferrostatin-1 (Fer-1), which prevents lipid hydroperoxide formation, at a concentration of 5 μM. The following NO donors were used: diethylamine NONOate sodium salt hydrate (DEA NONOate), with a half-life of 2 min at 37°C and 15 min at 22–25°C, and dipropylenetriamine NONOate (DPTA NONOate), with a half-life of 3 h at 37°C and 5 h at 22–25°C (0.1 M phosphate buffer, pH 7.4). The first addition of NO donors was performed within 5–15 min after the ferroptosis induction; then the reagent was added at defined time intervals.

### Cell death characterization

Cell death was quantitatively assessed using the three independent methods.

1. Lactate dehydrogenase (LDH) release into the culture medium was measured using a standard LDH detection kit (Thermo Fisher Scientific, USA). 50 μl of reaction buffer was added to 50 μl of culture medium (control medium being a cell culture medium not incubated with macrophages) and incubated for 30 min. After adding 50 μl of the stop reagent, the absorbance of the solution was measured at 490 and 680 nm. The percentage of cell death was calculated as (LDH_sample_/ LDHcontrol)·100%.
2. Alamar blue reagent was added to the cells at a concentration of 10 μg/ml, followed by incubation at 37°C for 2 h. Fluorescence was measured using a VICTOR Nivo spectrofluorometer (PerkinElmer, USA), Ex — 580/20 nm, Em — 625/20 nm.
3. LIVE/DEAD Viability/Cytotoxicity Assay Kit (Thermo Fisher Scientific, USA) is a standard cell viability assay which allows to determine live from dead cells via simultaneous staining with green-fluorescent calcein AM and red-fluorescent propidium iodide homodimer-1. After replacing the medium, reagents of the LIVE/DEAD assay kit and 1 μM Hoechst were added for nuclear staining. The staining was conducted for 30 min at 37°C and 5% CO_2_ according to the manufacturer’s protocol, followed by medium replacement. Live and dead cells were visualized using an EVOS M5000 fluorescence microscope (Thermo Fisher Scientific, USA) with a following established wavelength range for excitation and registration of dye fluorescence: for Hoechst 33342 (Ex_max_ — 351 nm, Em_max_ — 461 nm) the DAPI channel was used (excitation at a wavelength of 357±44 nm, emission was registered at a range of 447±30 nm); for calcein AM (Ex_max_ — 494 nm, Em_max_ — 517 nm) the GFP channel was used (excitation at a wavelength of 470±22 nm, emission was registered at a range of 525±25 nm); for propidium iodide (Ex_max_ — 535 nm, Em_max_ — 617 nm) the RFP channel was used (excitation at a wavelength of 531±40 nm, emission was registered at a range of 593±20 nm).

### Visualization of lipid peroxidation in cells using the BODIPY lipid peroxidation sensor

THP-1 cells were seeded in 48-well plates, and 24 h after medium replacement, the BODIPY 581/591 C11 probe (Invitrogen, USA) was added to cells at a concentration of 15 μM. The incubation was at 37°C and 5% CO_2_. After 30 min, the ferroptosis inducer RSL3 was added to the experimental cells at a concentration of 1.25 μM. After 17–19 h, the medium was replaced, and samples were analyzed using the EVOS M5000 microscope. The probe integrating into the cell membrane exhibits red fluorescence (Ex_max_ — 581 nm, Em_max_ — 591 nm; GFP channel); upon oxidation, the fluorescence shifts to the green spectrum (Ex_max_ — 488 nm, Em_max_ — 510 nm; RFP channel).

### Statistical analysis

Statistical analysis was performed using the GraphPad Prism program version 9.0.5. A significance threshold of p<0.05 was established. Groups were compared using a two-way analysis of variance (two-way ANOVA) and one-way ANOVA followed by Tukey’s post-hoc test.

## Results

The ferroptosis inducers RSL3 or ML-162 were added to THP-1 macrophage culture medium at different concentrations of 0.5, 1.25, and 2.5 μM. Cell nativity was monitored using a light microscope and three biochemical methods: 1) the most accurate parameter that allows quantitative characterization of cell death is the concentration of LDH enzyme released from dead cells into the incubation medium; 2) a simple and visual method based on the use of the Alamar blue reagent allows us to assess the metabolic activity of living cells, which are able to reduce Alamar blue (resazurin) to fluorescent resorufin; 3) the LIVE/DEAD assay based on two dyes allows to visually distinguish between live and dead cells.

**Figure 1.**
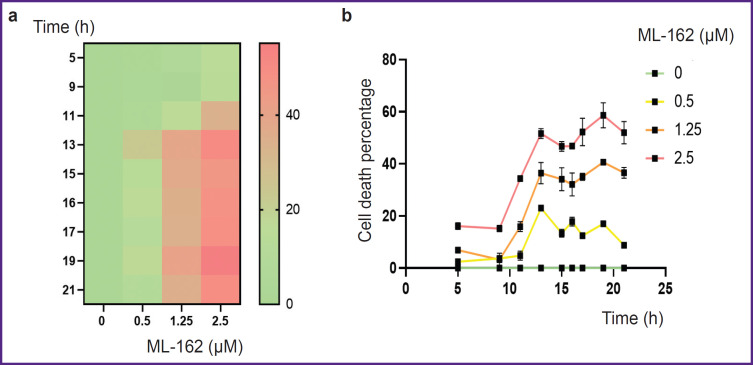
Dependence of ferroptosis in THP-1 macrophages on ML-162 inducer concentration and incubation time: (a) heatmap of ferroptosis development over time for different ML-162 inducer concentrations; (b) dependence of the cell death percentage on incubation time for three ML-162 concentrations

The time of ferroptosis development and the number of dead cells depend on the concentration of ferroptosis inducers and incubation time. Figure 1 shows that the level of LDH in the THP-1 macrophage medium increased with the increase in the concentration of ferroptosis inducer and the cell incubation time with the inducer. Under our experimental conditions, cell death starts after 5–9 h of incubation and reaches its maximum after 13–19 h depending on the inducer concentration.

**Figure 2.**
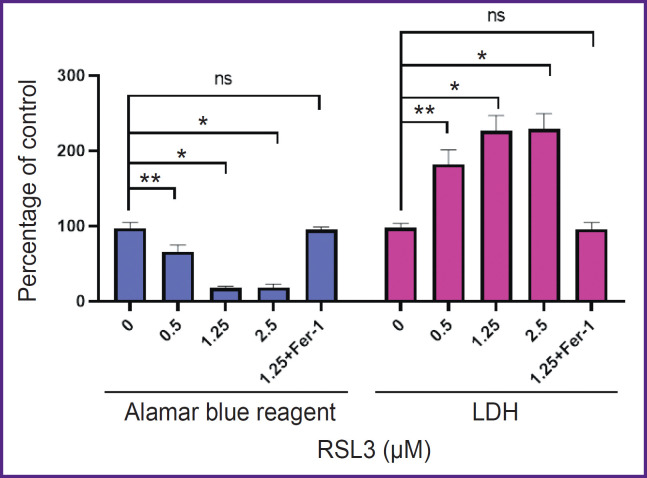
Quantitative characteristic of RSL3-induced ferroptosis in THP-1 cells using Alamar blue and lactate dehydrogenase (LDH) measurement in cell culture medium The incubation time with the inducer is 19 h. Fer-1, a specific ferroptosis inhibitor, completely inhibits cell death at a concentration of 5 μM; * p<0.0001, ** p<0.0005, ^ns^p>0.05

Figure 2 shows the results of an experiment comparing cell death measured with LDH and Alamar blue assays. An increase in LDH concentration was accompanied by a decrease in the number of live cells able to reduce resazurin. In this experiment, there was observed a good agreement between the results obtained by the two independent methods. However, in several other experiments, the results with Alamar blue did not accurately reflect cell viability (results not shown), likely due to macrophage oxidative activity or their release into medium because of death initiation. Therefore, the LDH-based method was chosen as the main method for quantitative characterization of lipid peroxidation.

**Figure 3.**
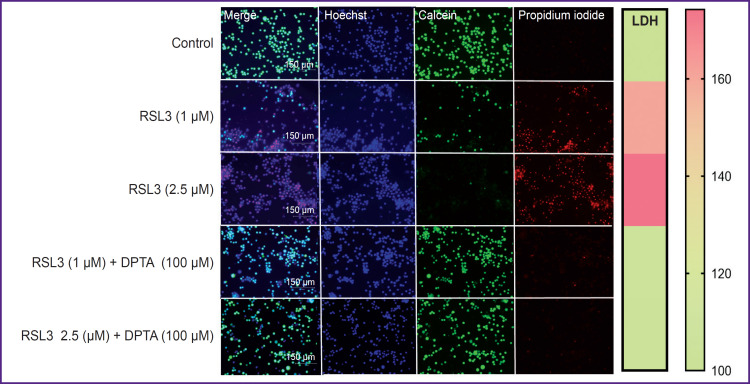
Microphotographs of THP-1 after additions of LIVE/DEAD assay reagents obtained using an EVOS M5000 fluorescence microscope (20×) The green channel is calcein (Ex — 488 nm), the red channel is propidium iodide (Ex — 561 nm), the blue channel is Hoechst (Ex — 405 nm). After 17 h of cell cultivation with ferroptosis inducer, a mixture of LIVE/DEAD assay dyes and 1 μM Hoechst (nuclei staining) was added to the cells. After 30 min of incubation, images of the cells were obtained: the respective additions to the wells are indicated next to the images. Results of lactate dehydrogenase (LDH) measurements in samples are shown on the right (relative units)

Cell death was visualized using the LIVE/DEAD assay (Figure 3). In the control, cells were stained green; live cells reduced calcein AM to fluorescent calcein. The addition of the ferroptosis inducer RSL3 dose- dependently decreased the number of live cells and increased the number of cells stained with propidium iodide, which can only penetrate dead cells.

**Figure 4.**
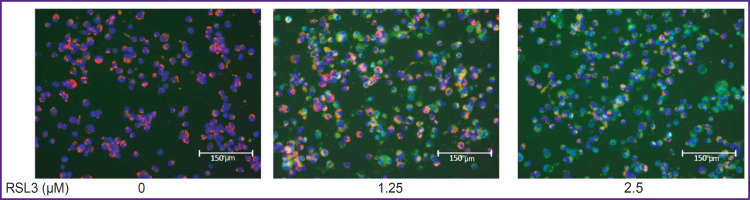
Visualization of lipid oxidation in THP-1 macrophages treated with different RSL3 concentrations using the lipid peroxidation sensor BODIPY C11 Incubation for 17 h, 20×. BODIPY fluorescence was recorded in the red spectral region after excitation at 561 nm and in the green spectral region after excitation at 488 nm; 1 μg/ml Hoechst was used to stain nuclei

To prove the ferroptotic form of cell death, we used a specific ferroptosis inhibitor Fer-1 (5 μM), which completely inhibited cell death (see Figure 2). Cell membrane lipid oxidation is a hallmark of ferroptosis. To additionally confirm that the cell death we studied was ferroptosis, we used the specific fluorescent lipid oxidation sensor BODIPY C11 (Figure 4). In our experiments, a dose-dependent change in BODIPY fluorescence was observed: there were a decrease in red fluorescence and an increase in green fluorescence with increasing RSL3 concentration.

**Figure 5.**
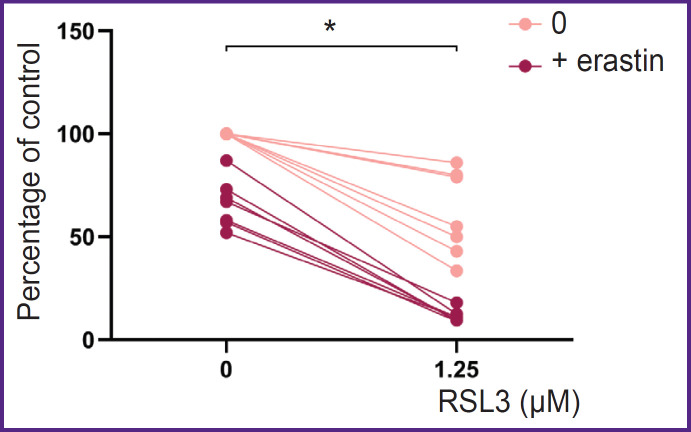
Comparison of the number of live THP-1 macrophages (percentage of control) incubated with RSL3 or with RSL3 + erastin Reagent concentrations: 1.25 μM RSL3 and 10 μM erastin; * p<0.0001

Erastin had little effect on the THP-1 macrophage viability; when 10 μM erastin was added, cell death was 23±7% of the control. Combined treatment with ferroptosis inducers of different mechanisms synergistically increased cell death (Figure 5).

**Figure 6.**
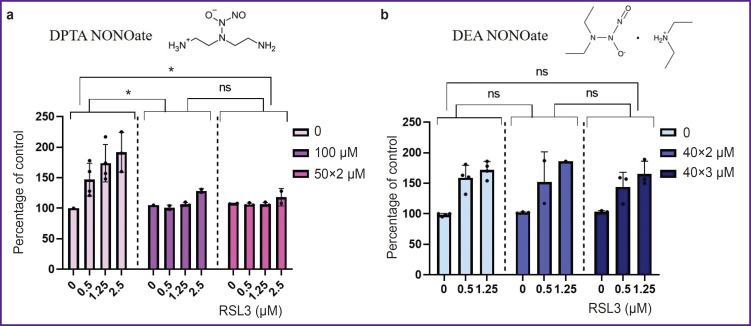
Lactate dehydrogenase concentration in the culture medium of THP-1 macrophages treated with ferroptosis inducer (RSL3) and NO donors with different half-lives (a) DPTA NONOate at concentrations of 100 μM (added once after the inducer) and 50 μM (added twice: after the inducer and after 4 h); (b) DEA NONOate was added several times at 40 μM at 3 h intervals. Incubation time was 17–19 h. Insets are chemical formulas of NO donors. * p<0.0001; ^ns^p>0.05

DPTA NONOate was used as a nitric oxide donor. DPTA completely inhibited ferroptosis in THP-1 cells at a concentration of 100 μM. Inhibition was observed regardless of whether the entire dose was added at once or split into two 50 μM additions (Figure 6 (a)). The NO donor effect on ferroptosis in THP-1 cells was also shown using the LIVE/DEAD assay (see Figure 3). The presence of 100 μM DPTA in the culture medium completely inhibited ferroptosis even at the maximum RSL3 concentration (2.5 μM) and no propidium-stained cells were detected.

The donor DEA NONOate was also used in the experiments. The donor was added after ferroptosis inducer 2–3 times at 40 μM with an interval of 3 h. DEA NONOate did not affect the development of ferroptosis at any concentration of the inducer (Figure 6 (b)).

## Discussion

Ferroptosis is a non-apoptotic process of cell death, with iron-dependent accumulation of lipid hydroperoxides being the fundamental feature. Ferroptotic cell death underlies a number of diseases, such as neurodegenerative diseases, liver and cardiac injury during ischaemia-reperfusion, and others [5, 25]. Therefore, the search for ferroptosis inhibitors with therapeutic potential for ferroptosis-associated pathologies is an important objective [16, 26].

The ferroptotic form of cell death has been shown for many types of cells, but it remains poorly studied in human macrophages. Most research has focused mainly on mouse macrophages [9, 20]. In the current study, we used THP-1 macrophages as a model of human macrophages [27]. Ferroptosis in THP-1 macrophages is a slow process, beginning approximately 5 h after the addition of the inducers RSL3 or ML-162. The degree of ferroptosis development depended on the dose of the added inducer and reached a maximum 17–19 h after the death program initiation (see Figure 1). The ferroptosis development was characterized by three independent methods: LDH measurement in conditioned cell culture medium, reduction of Alamar blue to resorufin by live cells, and the standard LIVE/DEAD assay with calcein AM and propidium iodide (see Figures 2 and 3). The results of different methods were in good agreement, but the simplest and most reliable was the LDH measurement. The specific ferroptosis inhibitor Fer-1 completely inhibited the action of ferroptosis inducers at a concentration of 5 μM. Additionally, evidence of ferroptotic cell death was obtained using the fluorescent lipid peroxidation sensor BODIPY C11. In the cell membrane, BODIPY fluoresces in the red spectral region, but upon extensive lipid oxidation, lipid radicals oxidize BODIPY, shifting fluorescence to the green spectrum in cells treated with the ferroptosis inducer. The number of cells showing green fluorescence depended on the dose of inducer added to the incubation culture medium (see Figure 4).

The results for two ferroptosis inducers, RSL3 and ML-162, both being GPX4 inhibitors, did not differ; ferroptosis induction in THP-1 cells was already observed at a reagent concentration of 0.5 μM. Erastine, being the cystine/glutamate system inhibitor, reducing cellular GSH concentration, is a weak ferroptosis inducer for THP-1 macrophages, in contrast to GPX4 inhibitors. This has also been shown previously for RAW 264.7 macrophages [20]. Cell death upon addition of 10 μM erastin was 23±11% of control. However, simultaneous addition of the two types of inducers resulted in increased ferroptosis. The effect was not additive (see Figure 5).

It has previously been shown that resistance of proinflammatory M1 RAW 264.7 macrophages to ferroptosis is due to iNOS expression and, consequently, NO production [20]. Nitric oxide can be added to cells in the form of NO donors, i.e., substances that release gaseous NO when exposed to culture medium with pH 7.4. The authors used NO donors with a long half-life, such as DPTA (3 h at 37°С) and Diethylenetriamine NONOate (20 h at 37°С), while the time of ferroptosis development was 5 h. Adding NO donors to mouse macrophages increased their resistance to ferroptosis. Since human macrophages produce low levels of NO [28], we have set out to study how exogenous NO donors affect ferroptosis in THP-1 macrophages.

We compared the effects of donors with different halflives at 37°С — with a short half-life of DEA NONOate (2 min) and with a relatively long half-life of DPTA NONOate (3 h). Donors were added either once after the inducer at a concentration of 100 μM or repeatedly until the target concentration was reached. DEA NONOate had no effect on ferroptosis in THP-1 macrophages, whereas DPTA completely inhibited ferroptosis both when added once and when 50 μM was added twice. The rapid decomposition of DEA NONOate results in the fact that the produced NO apparently does not have time to enter the cells. Ferroptosis in THP-1 macrophages begins only ~5 h after the addition of the inducer. The complete inhibition of ferroptosis by a single DPTA addition (100 μM) at the beginning of the experiment suggests that the mechanism of NO action may be as follows: nitric oxide either modifies proteins participating in ferroptosis (e.g., interacts with iron in the active site of lipoxygenase-15) or is incorporated into iron dinitrosyl complexes with subsequent slow release [29].

## Conclusion

Three independent methods of cell viability assays have shown that GPX4 inhibitors dose-dependently induce cell death in THP-1 macrophages. Death inhibition by a specific Fer-1 inhibitor along with detection of cell membrane lipid oxidation by the fluorescent sensor BODIPY C11 prove that cells die through a programmed ferroptotic form of death. Simultaneous treatment of cells with ferroptosis inducers with different mechanism of action enhances ferroptosis. This study shows that NO produced by DPTA NONOate with a half-life of 3 h inhibits ferroptosis in macrophages, which develops around 17–19 h. Therefore, there are mechanisms for prolonging the action of NO in cells. The study of mechanisms of ferroptosis inhibition by NO donors is important for further use of these compounds for the regulation of ferroptosis-associated pathologies.
